# Downregulation of PPARα during Experimental Left Ventricular Hypertrophy is Critically Dependent on Nox2 NADPH Oxidase Signalling

**DOI:** 10.3390/ijms21124406

**Published:** 2020-06-20

**Authors:** Adam P. Harvey, Emma Robinson, Kevin S. Edgar, Ross McMullan, Karla M. O’Neill, Matthew Alderdice, Raheleh Amirkhah, Philip D. Dunne, Barbara J. McDermott, David J. Grieve

**Affiliations:** 1 Wellcome-Wolfson Institute for Experimental Medicine, Queen’s University Belfast, Belfast BT9 7AE, UK; adam.harvey@stryker.com (A.P.H.); emmarobinsonscott@gmail.com (E.R.); k.edgar@qub.ac.uk (K.S.E.); rmcmullan07@qub.ac.uk (R.M.); karla.oneill@qub.ac.uk (K.M.O.); b.mcdermott@qub.ac.uk (B.J.M.); 2 Patrick G Johnston Centre for Cancer Research, Queen’s University Belfast, Belfast BT7 1NN, UK; m.alderdice@qub.ac.uk (M.A.); r.amirkhah@qub.ac.uk (R.A.); p.dunne@qub.ac.uk (P.D.D.)

**Keywords:** Nox2 NADPH oxidase, PPARα, left ventricular hypertrophy, oxidative stress, microarray, transverse aortic constriction, chronic heart failure

## Abstract

Pressure overload-induced left ventricular hypertrophy (LVH) is initially adaptive but ultimately promotes systolic dysfunction and chronic heart failure. Whilst underlying pathways are incompletely understood, increased reactive oxygen species generation from Nox2 NADPH oxidases, and metabolic remodelling, largely driven by PPARα downregulation, are separately implicated. Here, we investigated interaction between the two as a key regulator of LVH using in vitro, in vivo and transcriptomic approaches. Phenylephrine-induced H9c2 cardiomyoblast hypertrophy was associated with reduced PPARα expression and increased Nox2 expression and activity. Pressure overload-induced LVH and systolic dysfunction induced in wild-type mice by transverse aortic constriction (TAC) for 7 days, in association with Nox2 upregulation and PPARα downregulation, was enhanced in PPARα^−/−^ mice and prevented in Nox2^−/−^ mice. Detailed transcriptomic analysis revealed significantly altered expression of genes relating to PPARα, oxidative stress and hypertrophy pathways in wild-type hearts, which were unaltered in Nox2^−/−^ hearts, whilst oxidative stress pathways remained dysregulated in PPARα^−/−^ hearts following TAC. Network analysis indicated that Nox2 was essential for PPARα downregulation in this setting and identified preferential inflammatory pathway modulation and candidate cytokines as upstream Nox2-sensitive regulators of PPARα signalling. Together, these data suggest that Nox2 is a critical driver of PPARα downregulation leading to maladaptive LVH.

## 1. Introduction

Chronic heart failure (CHF) represents a major cause of morbidity and mortality in the Western world. Although the molecular mechanisms underlying the development and progression of this devastating disease are incompletely understood, it is well established that reactive oxygen species (ROS) derived from the NADPH oxidase (Nox) enzymes are a critical mediator of adverse cardiac remodelling [1,2,3]. The prototypic Nox, Nox2, comprises a catalytic core (Nox2/p22^phox^), together with several cytosolic subunits (p47^phox^, p67^phox^, p40^phox^, Rac) that associate upon activation. Seven Nox isoforms have been identified (Nox1–5 and Duox1–2), with Nox2 and Nox4 being highly expressed in the heart with specific localisation in cardiomyocytes, endothelial cells and fibroblasts. The primary function of these enzymes is to generate ROS by catalysing electron transfer from NADPH to molecular oxygen. In phagocytes, Nox2 mediates microbicidal activity through generation of large amounts of superoxide, whilst in non-phagocytic cells, Nox proteins generate low levels of ROS that are involved in intracellular signalling [4]. Previous studies have established that Nox2 is critically involved in the development and progression of cardiac hypertrophy and contractile dysfunction in response to various stimuli, including angiotensin II, pressure overload, and myocardial infarction [3,5,6]. Notably, within the myocardium, Nox2-derived ROS target numerous cellular compartments, organelles and signalling cascades to exert diverse effects and thereby play a critical role in key remodelling processes, including cardiomyocyte hypertrophy [7,8,9]. Whilst it is clear that ROS are important in the pathogenesis of CHF, clinical antioxidant trials have produced disappointing results, highlighting the requirement for more detailed understanding of their specific function and targets in the myocardium and the rationale for selective therapeutic targeting of individual ROS species and/or sources [10].

In addition to the established involvement of Noxs, downregulation of peroxisome proliferator-activated receptor-α (PPARα) is also recognised as an important mediator of pressure overload-induced left ventricular hypertrophy (LVH). This appears to be largely due to the characteristic phenotypic switch in metabolic regulation within the failing myocardium, whereby fatty acid oxidation is no longer the predominant pathway and is replaced by a reliance on glucose/lactate utilisation [11]. Indeed, PPARα is the most abundant PPAR isoform in cardiac tissue, where its activation leads to increased expression of numerous genes involved in important fatty acid oxidation processes [12,13], underlying its key function in regulation of cardiac metabolic substrate preference [13,14,15]. Notably, a number of experimental studies have indicated that PPARα activation exerts beneficial effects on LVH development and progression. Initial reports of downregulation of PPARα following aortic banding in mice [12] were corroborated in PPARα^−/−^ mice, which demonstrated an enhanced hypertrophic response to pressure overload [15]. These effects were associated with differential expression of genes related to oxidative stress, immune response, extracellular matrix remodelling and inflammatory pathways, thereby highlighting an important role beyond its well characterised effects on cardiac metabolism [16]. Importantly, PPARα downregulation is also observed in clinical hypertrophic CHF [17,18], although it should be noted that increased PPARα expression is reported in human dilated cardiomyopathy [19,20], highlighting potential stress-dependent differences in cardiac PPARα signalling. Consistent with PPARα downregulation in hypertrophic CHF and the worsened phenotype observed in PPARα^−/−^ mice, numerous experimental studies have reported beneficial effects of PPARα activation in this setting. For example, fenofibrate attenuated pressure overload-induced LVH in rodent models [21,22,23], whilst chronic treatment with PPARα agonists protected against hypertensive cardiac hypertrophy [24,25,26]. Similarly, PPARα overexpression reduced phenylephrine (PE)-induced hypertrophy in rat neonatal cardiomyocytes [27], whilst PPARα activation protected against endothelin-1- and angiotensin II-induced hypertrophy in isolated rat cardiomyocytes [23,24,28,29], consistent with a cardioprotective role.

Whilst it is well established that both PPARα and NADPH oxidases play important roles during cardiac remodelling, emerging evidence suggests that cross-talk or interaction between these two proteins may represent a key regulatory axis in CHF by directing opposing actions on central signalling pathways. Indeed, it is reported that suppression of NADPH oxidase-derived ROS worsens acute myocardial ischaemia/reperfusion injury through a combination of PPARα upregulation and HIF-1α downregulation [30], thereby providing support for an important relationship between these two proteins in the context of pressure overload-induced LVH. Interestingly, downregulation of PPARα and upregulation of Noxs, particularly Nox2, play critical and independent roles in cardiac hypertrophy [6,12,31], and these effects often manifest as opposing actions on key signalling pathways, such as Akt, AP-1, NF-κB and MAPKs, which are differentially regulated by PPARα and Nox2. We, therefore, hypothesise that these intracellular signalling pathways represent key linkage points between PPARα and NADPH oxidases, which is supported by emerging evidence [32]. Although a key relationship between PPARα and NADPH oxidases has been suggested, the precise nature of such interaction and its implications for LVH development remain unclear. To date, several studies have provided evidence supporting a negative co-regulatory relationship between Noxs and PPARα [33]. For example, increased expression of the Nox subunits, p22^phox^ and p47^phox^, and decreased antioxidant expression (e.g., catalase, superoxide dismutase) are observed in PPARα^−/−^ mice [34]. Indeed, it is well established that Noxs regulate cellular metabolism in many diverse systems [35], so it possible that PPARα activation may inhibit ROS signalling and induction of downstream redox-sensitive transcription factors, with resultant cardioprotection, whilst upregulation of cardiac Noxs may lead to downregulation of PPARα with consequent detrimental actions. The aim of this study was, therefore, to investigate the specific relationship between Nox2 NADPH oxidase-derived ROS and PPARα during development of LVH and contractile dysfunction, and to identify key mechanisms of interaction, using an established experimental model of pressure overload associated with LVH and systolic dysfunction [36] as key factors underlying progression towards hypertrophic CHF.

## 2. Results

### 2.1. H9c2 Cardiomyoblast Hypertrophy and Associated NADPH Oxidase-Dependent Superoxide Production Are Inhibited by PPARα Activation

**Figure 1:**
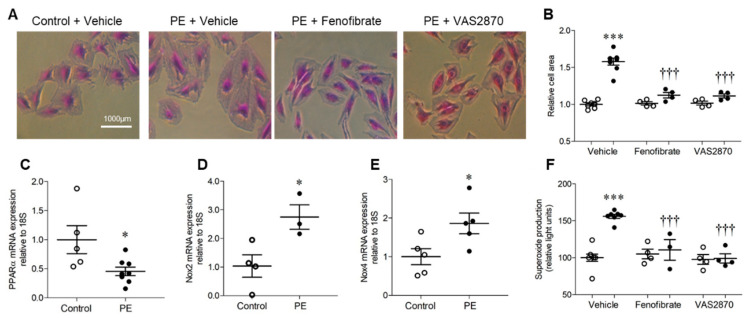
H9c2 cardiomyoblast hypertrophy and associated NADPH oxidase-dependent superoxide production are inhibited by PPARα activation. (**A**,**B**) H9c2 cells stimulated with phenylephrine (PE) display hypertrophy as indicated by increased cell area, which is prevented by PPARα activation (fenofibrate, 10 µmol/L) and NADPH oxidase inhibition (VAS2870, 10 µmol/L). Representative images (10× magnification) are shown in (**A**) and mean data in (**B**). (**C**) PE induces downregulation of PPARα mRNA and upregulation of (**D**) Nox2 and (**E**) Nox4 mRNA expression. (**F**) PE stimulation is associated with elevated NADPH-dependent superoxide production with is abolished by PPARα activation or NADPH oxidase inhibition. Mean ± SEM; *N* = 3–8; * *p* < 0.05, *** *p* < 0.001 vs. control, ^†††^ and *p* < 0.001 vs. PE alone.

Treatment of H9c2 cardiomyoblasts with 10 μmol/L PE for 24 h resulted in significant hypertrophy, as indicated by ~50% increase in cell area, which was prevented by both PPARα activation with fenofibrate and Nox inhibition with VAS2870 (Figure 1A,B). This finding is consistent with the established role of PPARα downregulation in mediating the metabolic switch from myocardial fatty acid oxidation to glucose/lactate utilisation during cardiomyocyte hypertrophy, and the key involvement of Nox-derived ROS [6,7,15,16]. Indeed, PE-induced H9c2 hypertrophy was associated with decreased expression of PPARα mRNA and increased expression of Nox2 and Nox4 mRNA (Figure 1C,E), indicating differential regulation in this setting. Furthermore, elevated levels of superoxide observed in response to PE were independently prevented by VAS2870 and fenofibrate (Figure 1F), indicating suppression by PPARα activation and Nox dependency. Taken together, these initial observations support our hypothesis that interaction between PPARα and Nox2 may represent an important driver of cardiomyocyte hypertrophy.

### 2.2. Pressure Overload-induced Cardiac Hypertrophy is Dependent upon Both PPARα and Nox2

**Figure 2:**
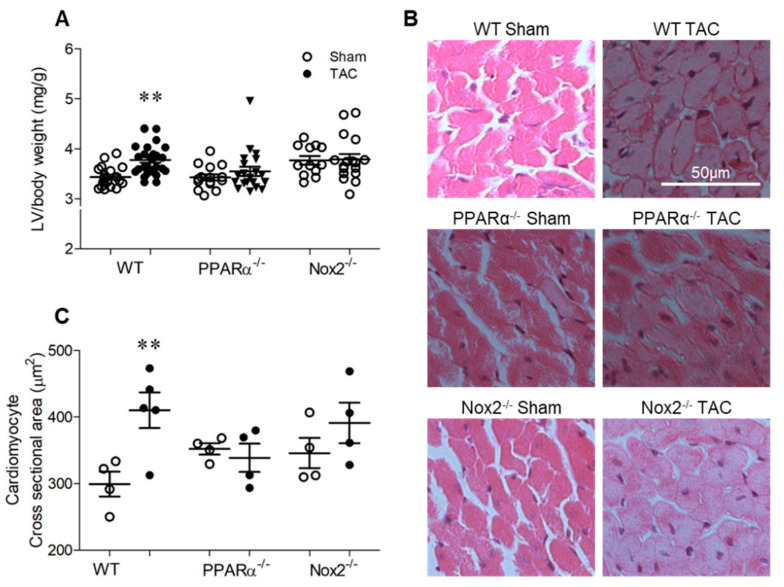
Pressure overload-induced cardiac hypertrophy is dependent upon PPARα and Nox2. (**A**) Hypertrophic increases in LV weight normalised to body weight following pressure overload in WT animals are not evident with PPARα^−/−^ or Nox2^−/−^ (*N* = 12–28). (**B**) Representative images of LV sections stained with haematoxylin and eosin with (**C**) quantification of mean cardiomyocyte cross-sectional area (*N* = 4–5). Mean ± SEM; ** *p* < 0.01 vs. corresponding sham.

TAC-induced pressure overload resulted in a significant increase in LV/body weight ratio in wild-type (WT) mice of ~11% after 7 days, which was not evident in either PPARα^−/−^ or Nox2^−/−^ mice (Figure 2A). Similarly, analysis of cardiomyocyte cross-sectional area in LV sections indicated significant cell hypertrophy in WT TAC hearts of ~37%, which was absent in both PPARα^−/−^ and Nox2^−/−^ hearts subjected to pressure overload (Figure 2B,C). Of note, both fenofibrate-mediated PPARα activation in vitro (Figure 1) and genetic deletion of PPARα in vivo (Figure 2) prevented PE- and TAC-induced cardiomyocyte hypertrophy, respectively, which was inhibited by either pharmacological (Figure 1) or genetic perturbation (Figure 2) of Nox2, respectively.

### 2.3. Pressure Overload-induced Cardiac Dysfunction is Nox2 Dependent and Worsened in the Absence of PPARα

**Figure 3:**
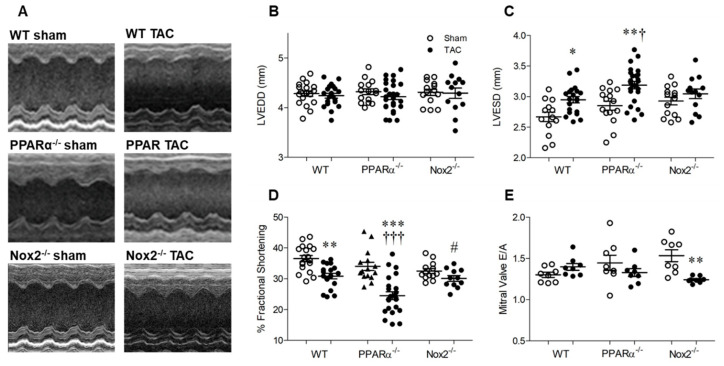
Pressure overload-associated cardiac dysfunction is Nox2 dependent and worsened in the absence of PPARα. (**A**) Representative echocardiography M-mode images from WT, PPARα^−/−^ and Nox2^−/−^ mice subjected to TAC or sham operation. (**B**) Left ventricular end-diastolic diameter (LVEDD) is unchanged by pressure overload, whilst (**C**) left ventricular end-systolic diameter (LVESD) is increased by pressure overload in WT animals, an effect which is amplified in the absence of PPARα and prevented in the absence of Nox2. (**D**) Percentage fractional shortening is decreased by pressure overload in WT animals and further reduced in the absence of PPARα and prevented in the absence of Nox2. (**E**) Mitral valve E/A ratio is decreased by pressure overload in the absence of Nox2 but unaltered in WT and PPARα^−/−^ animals. Mean ± SEM; *N* = 11–26; * *p* < 0.05, ** *p* < 0.01, *** *p* < 0.001 vs. corresponding sham, ^†^ *p* < 0.01, ^†††^ *p* < 0.001 vs. corresponding WT, and ^#^ *p* < 0.05 vs. corresponding PPARα.

Echocardiography data are presented in Figure 3. Heart rate remained similar between groups (WT sham: 454 ± 15, WT TAC: 493 ± 15, PPARα^−/−^ sham: 453 ± 0.10, PPARα^−/−^ TAC: 485 ± 12, Nox2^−/−^ sham: 453 ± 0.10, Nox2^−/−^ TAC: 485 ± 12 bpm; *n* = 13−26, *p* = NS). LV end-diastolic diameter (LVEDD) was also unaltered in WT TAC mice, indicating that LV chamber dilatation was not evident at 7 days, and was unaffected by genotype (Figure 3B). Interestingly, whilst genetic deletion of PPARα or Nox2 was protective against TAC-induced LVH, associated systolic dysfunction, as indicated by increased LV end-systolic diameter (LVESD) and reduced fractional shortening in WT mice, was differentially altered (Figure 3C,D). In PPARα^−/−^ mice subjected to TAC, LVESD was further increased whilst fractional shortening was markedly reduced compared to WT controls, indicating significantly worsened systolic dysfunction. In contrast, systolic function was preserved in Nox2^−/−^ TAC mice. Diastolic dysfunction (which was not present in WT or PPARα TAC mice) was evident in these animals, as reflected by reduced mitral valve E/A ratio (Figure 3E), suggesting the delayed progression of cardiac contractile dysfunction.

### 2.4. Pressure Overload is Associated with Altered Myocardial Redox Status and PPARα Expression

**Figure 4:**
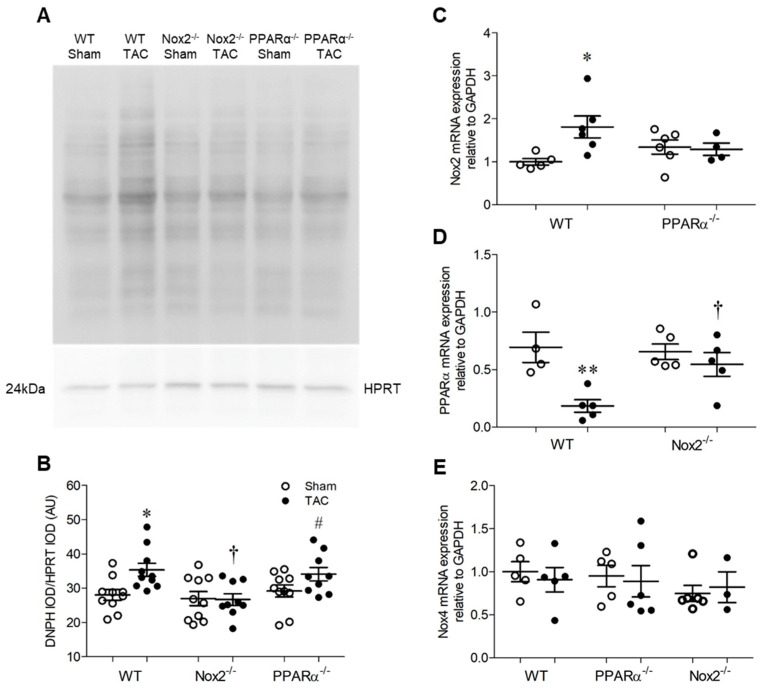
Pressure overload is associated with altered myocardial redox status and PPARα expression. (**A**,**B**) Myocardial protein carbonylation is elevated in WT animals following pressure overload, an effect that is prevented in the absence of Nox2 but unaltered in the absence of PPARα. Representative blot is shown in (**A**) and mean data in (**B**) (*N* = 8–10). (**C**) Nox2 transcript levels are elevated by pressure overload in hearts of WT animals but normalised in the absence of PPARα. (**D**) PPARα transcript levels are reduced by pressure overload in hearts of WT animals but normalised in the absence of Nox2. (**E**) Nox4 transcript levels are unchanged by pressure overload (*N* = 3–6). Mean ± SEM; * *p* < 0.05, ** *p* < 0.01 vs. corresponding sham, ^†^ *p* < 0.05 vs. corresponding WT, and ^#^ *p* < 0.05 vs. corresponding Nox2^−/−^.

Pressure overload-induced LVH was associated with increased levels of oxidised proteins in hearts from WT TAC mice, as assessed by Oxyblot assay (Figure 4A,B). However, this pattern was absent in Nox2^−/−^ TAC mice and unaltered in PPARα^−/−^ TAC mice, indicating that oxidative changes observed in WT mice were critically-dependent upon Nox2, but occurred independently of PPARα signalling. This may, in part, explain why Nox2^−/−^ TAC mice were protected against development of systolic dysfunction, whilst PPARα^−/−^ TAC mice, in which detrimental redox alterations were still evident, demonstrated further impaired cardiac function. Nonetheless, as anticipated, adverse functional changes observed in WT mice were associated with differential regulation of Nox2 and PPARα, whose expression was increased and decreased, respectively, in response to TAC (Figure 4C,D). Interestingly, upregulation of Nox2 observed in WT TAC mice was prevented by PPARα deletion whilst downregulation of PPARα was not evident in the absence of Nox2 (Figure 4C,D), further highlighting the potential for important interaction between these two proteins as a key regulator of cardiac function. Notably, whilst Nox2 expression was increased in response to TAC in a PPARα-dependent manner, LV Nox4 mRNA levels remained unaltered by TAC across all three genotypes (Figure 4E), indicating that Nox isoform cross-regulation, which is reported in other settings [2,5], does not seem to play a significant role in response to pressure overload.

### 2.5. Nox2 and PPARα Deletion Result in Significant Transcriptomic Alterations in Pathways Mediating PPARα Signalling and Response to Oxidative Stress in Mouse Cardiac Tissue

**Figure 5:**
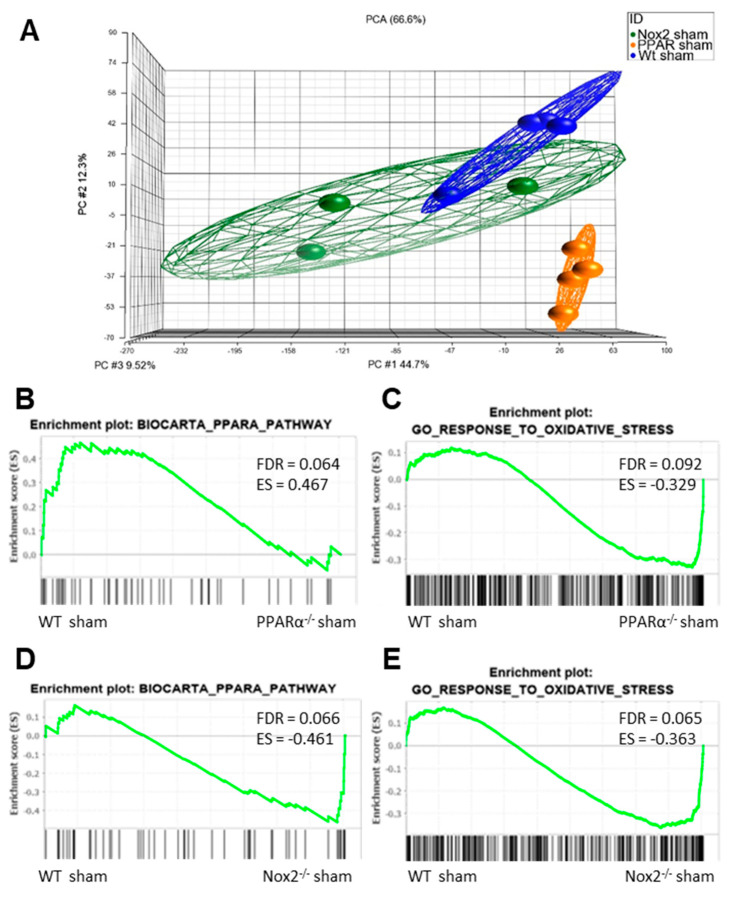
Nox2 and PPARα deletion result in significant transcriptomic alterations in pathways mediating PPARα and oxidative stress signalling in mouse cardiac tissue. (**A**) Principal component analysis demonstrating altered clustering of transcript expression in WT, PPARα^−/−^, and Nox2^−/−^ sham hearts. GSEA plots depicting cardiac transcript expression in (**B**) Biocarta PPARα pathway and (**C**) GO response to oxidative stress in WT sham vs. PPARα^−/−^ sham, and (**D**) Biocarta PPARα pathway and (**E**) GO response to oxidative stress in WT sham vs. Nox2^−/−^ sham. *N* = 3–4.

In order to determine whether significant interaction between Nox2 and PPARα occurs in an in vivo setting, an extensive transcriptomic analysis of LV tissue isolated from sham-operated WT, PPARα^−/−^ and Nox2^−/−^ mice was performed. Principal component analysis initially demonstrated distinct clustering of cardiac tissue on the basis of experimental group, highlighting significant transcriptomic variation (Figure 5A). Furthermore, gene set enrichment analysis (GSEA) (37) indicated overlapping alterations in PPARα and oxidative stress pathways, with specific repression (FDR < 0.25) of genes in the Biocarta PPARα pathway and elevation of those in the GO response to oxidative stress in hearts from PPARα^−/−^ sham mice versus WT sham mice, whilst genes in both pathways were elevated in Nox2^−/−^ sham versus WT sham hearts. This initial observation of alteration of genes in these key biological pathways between genotypes with differential directionality provides clear evidence of overlapping cardiac signalling cascades regulated by Nox2 and PPARα.

**Figure 6:**
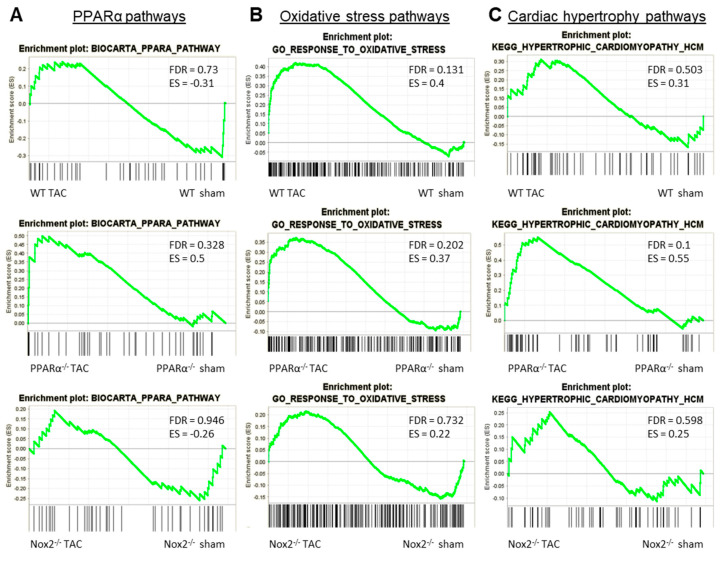
GSEA comparison of alterations in key pathways in WT, PPARα^−/−^ and Nox2^−/−^ mice subjected to pressure overload. Plots depicting expression of cardiac transcripts in sham vs. TAC mice across different genotypes: (**A**) Biocarta PPARα pathway, (**B**) GO response to oxidative stress, and (**C**) KEGG hypertrophic cardiomyopathy pathway. *N* = 3–4.

To further interrogate the nature of this apparent interaction between Nox2 and PPARα in the context of LVH development, GSEA comparison of alterations in three key pathways between genotypes in response to pressure overload was performed. Specifically, regulation of genes involved in Biocarta PPARα pathway, GO response to oxidative stress, and KEGG hypertrophic cardiomyopathy pathway were assessed in LV tissue isolated from WT, PPARα^−/−^ and Nox2^−/−^ mice subjected to TAC. WT animals displayed significant alterations (FDR < 0.25) in GO response to oxidative stress, with no alterations to Biocarta PPARα pathway or KEGG hypertrophic cardiomyopathy pathway (Figure 6A–C). Notably, all three pathways remained unaltered in LV tissue from Nox2^−/−^ mice subjected to TAC, whilst PPARα^−/−^ mice showed significant alterations to both GO response to oxidative stress and KEGG hypertrophic cardiomyopathy pathway (Figure 6B,C), consistent with the observed worsened LVH phenotype. Considering that Nox2 was found to be essential for PPARα downregulation following TAC (Figure 4D) and that GO response to oxidative stress remains dysregulated in PPARα^−/−^ mice following TAC (Figure 6B), it seems likely that the predominant directional relationship between Nox2 and PPARα in this setting is represented by Nox2-derived ROS-mediated regulation of PPARα signalling, which is consistent with a previous report in the setting of acute myocardial ischaemia/reperfusion injury [30].

### 2.6. Cardiac Transcriptomic Alterations Induced by Pressure Overload in WT Mice are Differentially Regulated in the Absence of Nox2 and PPARα and Are Critically Dependent upon Functional Nox2 Signalling

**Figure 7:**
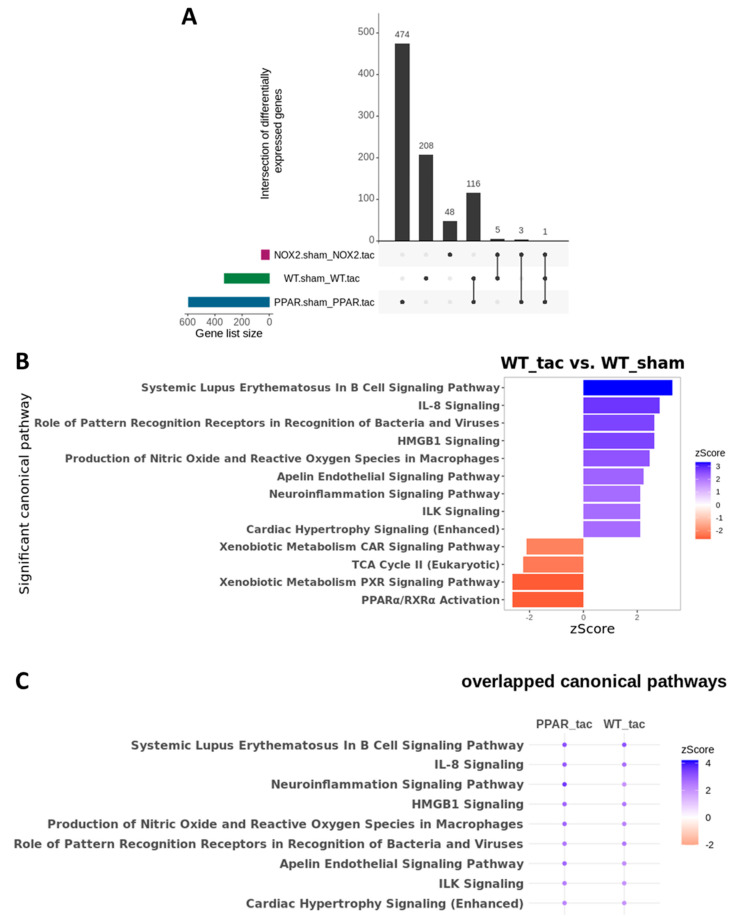
Cardiac transcriptomic analysis in WT, PPARα^−/−^ and Nox2^−/−^ mice subjected to pressure overload. (**A**) UpSet plot indicating intersection of differentially expressed genes between WT, Nox2^−/−^ and PPARα^−/−^ mice subjected to pressure overload (*N* = 3–4). (**B**) IPA was used to identify potential activated and inhibited canonical pathways with activation z-scores >2 (blue) or <−2 (red) in WT mice subjected to pressure overload. (**C**) Dot plot representing significant common canonical pathways with activation z-scores >2 (blue) or <−2 (red) in WT and PPARα^−/−^ mice subjected to pressure overload. All indicated pathways are statistically significant (–log(*p*-value) >1.3).

**Figure 8:**
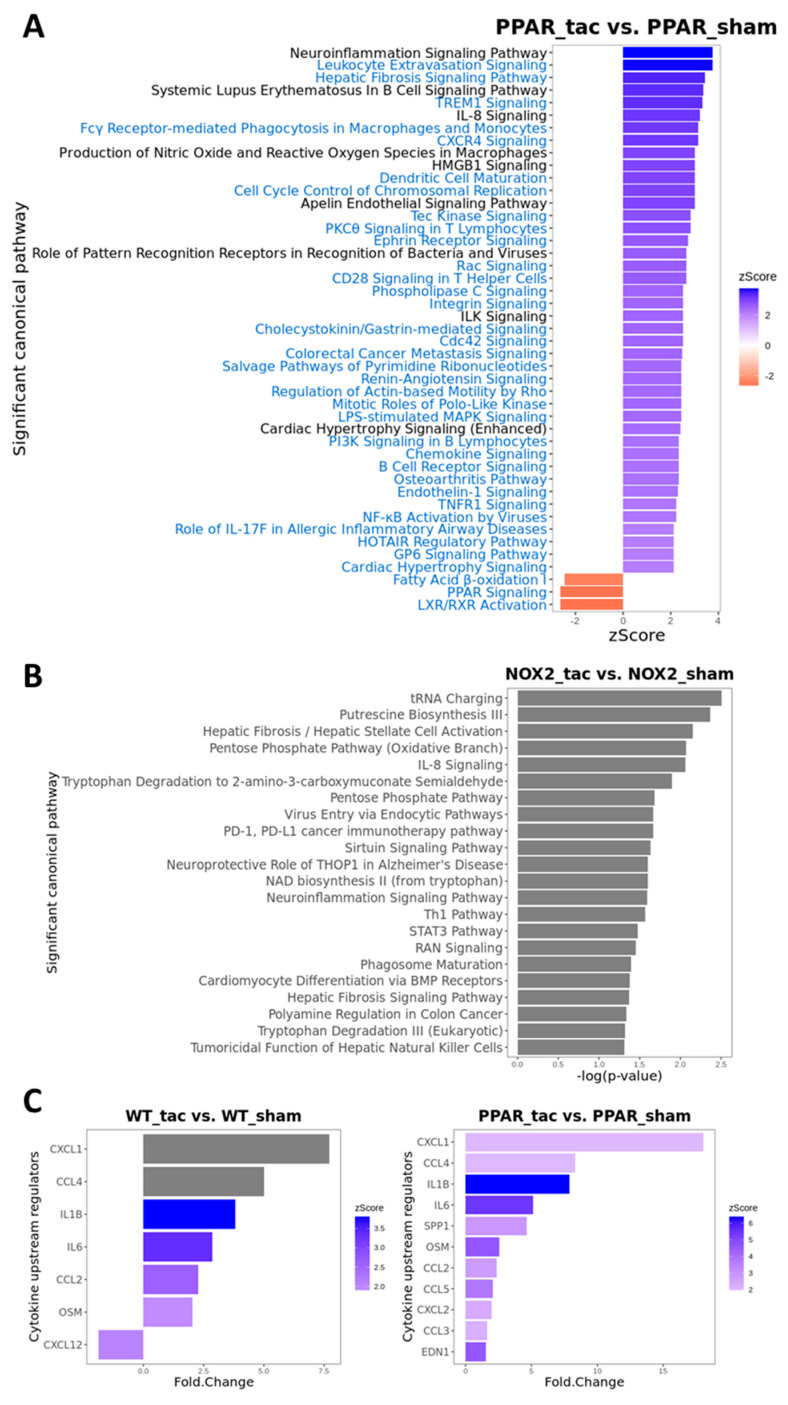
Canonical pathway analysis of differentially expressed genes using IPA. (**A**) Significantly enriched pathways in PPARα^−/−^ TAC vs. sham mice with activation z-scores >2 (blue) or <−2 (red). Pathway names highlighted in blue indicate unique enriched pathways in PPARα^−/−^ TAC vs. PPARα^−/−^ sham as compared to WT TAC vs. WT sham mice. (**B**) Significantly enriched pathways in Nox2^−/−^ TAC vs. sham mice (grey indicates that no activation z-score could be predicted by IPA). (**C**) Predicted cytokine upstream regulators associated with WT TAC vs. sham mice (left); and PPARα^−/−^ TAC vs. sham mice (right) with activation z-scores >2 (grey indicates no activation z-score). *N* = 3–4.

To test this hypothesis further, and to determine the specific influence of PPARα and Nox2 on cardiac transcriptomic alterations in response to pressure overload-induced LVH, differential gene expression analysis was next performed focussing on TAC-altered genes across the three genotypes. These analyses identified 208 differentially expressed genes (DEGs) in WT, 474 DEGs in PPARα^−/−^, and 48 DEGs in Nox2^−/−^ hearts following TAC (Figure 7A). Notably, >50% of gene expression changes observed in WT hearts were also differentially expressed in PPARα^−/−^ hearts (116 genes out of 208), suggesting conserved biological signalling in response to TAC. In contrast, there was limited overlap of only five genes between WT and Nox2^−/−^ hearts and three genes between PPARα^−/−^ and Nox2^−/−^ hearts, suggesting that biological signalling induced by TAC in Nox2^−/−^ hearts is distinct from that observed in either WT or PPARα^−/−^ hearts (Figure 7A). To further characterise the biological signalling induced in each genotype following TAC, ingenuity pathway analysis (IPA) was performed to investigate whether DEGs were associated with specific biological pathways. In WT hearts subjected to TAC, significant upregulation of ROS-dependent signalling associated with a number of immune, inflammatory, and hypertrophy-related pathways, was observed, in parallel with significant downregulation of metabolism-related CAR, PXR, and PPARα signalling (Figure 7B). Using the same approach to assess signalling changes in PPARα^−/−^ hearts subjected to TAC revealed similar overall activation and repression patterns compared to WT hearts (Figure 8A), with identical upregulation observed for nine pathways (Figure 7C). Taken together, these data suggest that while TAC was found to induce changes in PPARα pathways, PPARα itself does not appear to be an essential regular of these signalling cascades. In contrast, whilst Nox2^−/−^ hearts subjected to TAC were linked with a number of pathways identified from the DEG list, the fold-changes associated with these genes were not of sufficient magnitude to result in significant up- or downregulation of expression patterns (Figure 8C). As such, loss of significantly altered signalling in Nox2^−/−^ versus WT hearts suggests that Nox2 itself is an essential mediator of the TAC response, thereby supporting our conclusion that PPARα downregulation in pressure overload-induced LVH is dependent upon Nox2. Indeed, complementary inter-genotype comparisons revealed that metabolic pathways were primarily enriched in Nox2^−/−^ TAC versus WT TAC hearts (Figure S1), whilst upon evaluation of PPARα^−/−^ TAC versus WT TAC hearts, no DEGs were identified that met the required threshold, highlighting the apparent relative predominance of Nox2 with respect to PPARα in this setting.

### 2.7. Identification of Specific Mediators Underlying Directional Interaction between Nox2 and PPARα

**Figure 9:**
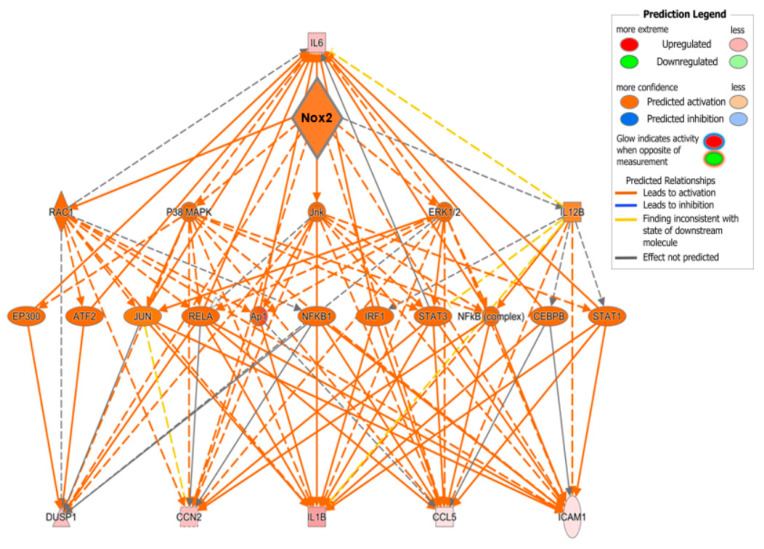
Predicted regulatory network influenced by Nox2 in PPARα^−/−^ mice subjected to pressure overload. IPA was used to generate the network. Node colour represents upregulated genes (red) and downregulated genes (green). Node shapes represent functional classes of gene products; rectangles for cytokines, triangles for phosphatases, concentric circles for groups or complexes, diamonds for enzymes, horizontal ovals for transcriptional regulators or modulators, and vertical oval for transmembrane receptor.

To examine potential mediators which may differentiate the predominantly observed changes in inflammatory pathways in WT and PPARα^−/−^ hearts, the resulting IPA data were used to predict cytokines that could act as upstream regulators associated with TAC response (Figure 8C). Notably, these analyses identified several cytokines (CXCL1, CCL4, IL1B, IL6, CCL2, and OSM) that were conserved between WT and PPARα^−/−^ hearts in response to TAC, suggesting that these inflammatory mediators regulate PPARα-independent response to pressure overload in LVH. In addition, focussed analysis in PPARα^−/−^ hearts identified several additional unique upstream cytokines (SPP1, CCL5, CXCL2, CCL3, EDN1) as potential PPARα-sensitive regulators of differentially activated pathways only observed in this genotype. Indeed, detailed network analysis of hearts from PPARα^−/−^ mice subjected to pressure overload (Figure 9) highlighted Nox2 as a predicted positive upstream regulator (z-score >2) of six genes (CCL5, CCN2, DUSP1, ICAM1, IL1B, and IL6) via a number of intermediate pathways (e.g., JNK, AP-1, and NF-κB) previously implicated in Nox2 signalling [37], providing further support for our conclusion that PPARα signalling in this setting is critical dependent upon Nox2. It is particularly interesting that three of the genes predicted to be regulated by Nox2 in response to TAC (IL1B, IL6, and CCL5) were also highlighted as likely upstream regulators of differentially activated pathways in WT and/or PPARα^−/−^ TAC hearts, indicating that these genes may represent central mediators of Nox2-dependent inflammatory signalling in pressure overload-induced LVH.

## 3. Discussion

Adverse cardiac remodelling in response to hypertension and ultimately leading to CHF is a significant global health burden. Despite extensive experimental studies investigating underlying mechanisms, advancement of novel therapeutic strategies to specifically target CHF are limited, with long-standing pharmacological treatments, such as ACE inhibitors and β-blockers, remaining as the standard approach to clinical management. In this regard, numerous experimental and clinical studies have identified critical but independent roles for Nox-derived ROS and PPARα in driving LVH and associated cardiac remodelling, highlighting these key signalling pathways as potential novel therapeutic targets. Indeed, in the current study, we specifically demonstrated concurrent myocardial upregulation of Nox2 and downregulation of PPARα with pressure overload-induced LVH. Furthermore, whilst genetic deletion of Nox2 protected against LVH and systolic dysfunction following TAC, deletion of PPARα was associated with worsened systolic function, despite lack of LVH when compared to WT mice. Intriguingly, specific interaction between Nox2 and PPARα was suggested by the observations that in vitro activation of PPARα prevented ROS production in H9c2 cardiomyoblasts, and dysregulated expression of PPARα and Nox2 in vivo did not occur with genetic deletion of Nox2 or PPARα, respectively. The combined finding that the presence of Nox2 was essential for downregulation of PPARα and that oxidative stress pathways remained dysregulated in PPARα^−/−^ mice subjected to TAC suggested highlighted Nox2-derived ROS-mediated PPARα downregulation as the predominant direction of interaction between these two key proteins. Indeed, differential gene expression analysis identified conserved biological signalling between WT and PPARα^−/−^ hearts in response to TAC, with limited overlap between these two genotypes and Nox2^−/−^ hearts subjected to TAC. In addition, IPA revealed similar overall activation and repression patterns of key immune, inflammatory, and hypertrophy-related pathways between WT and PPARα^−/−^ hearts which were not evident in Nox2^−/−^ hearts, suggesting that Nox2, but not PPARα, is not an essential regular of these TAC-modulated signalling cascades. Adding further support to our conclusion that Nox2-dependent regulation of PPARα signalling is the predominant direction of interaction in this setting, subsequent analysis, focussing on inflammatory pathways as established regulators of LVH [27], identified several PPARα-dependent and independent cytokines as likely mediators of TAC-modified pathways—some of which were strongly predicted to be regulated by upstream Nox2. As far as we are aware, this represents the first report of direct interaction between Nox2 and PPARα in the context of pressure overload-induced LVH with specific implication of inflammatory signalling and several candidate cytokine mediators which may be investigated as potential therapeutic targets.

It is well established that under pathological conditions, myocardial Nox2 activity and expression is increased and contributes significantly to CHF development and progression [2,5,33,38]. Specifically, Nox2-derived ROS are reported to be critically involved in several key cellular processes underlying cardiac remodelling, including interstitial fibrosis, MMP activation, cardiomyocyte hypertrophy, contractile dysfunction and apoptosis [39,40,41,42,43]. Notably, Nox2 is a key player in driving adverse remodelling associated with multiple pathological stimuli, as Nox2^−/−^ mice show reduced cardiac structural and functional alterations in response to, for example, experimental MI, angiotensin II infusion and doxorubicin administration, in comparison to WT controls [2,38,44]. In the present study, we confirm the previously reported key role for Nox2 in development of LVH and systolic dysfunction following pressure overload [6]. Whilst the role of Nox2 and other Nox isoforms in cardiac remodelling is complex, with differences in regulation, activation, subcellular localisation and ROS generation dictating distinct isoform-specific actions [45,46], a specific role for NADPH oxidases in regulating cardiac metabolism has been previously reported [47]. In this regard, in the present study we highlighted critical dependence of downregulation of the key metabolic regulator, PPARα, in response to TAC, on functional Nox2 signalling, suggesting an important role for this major cardiac NADPH oxidase isoform in regulating myocardial metabolic remodelling in this setting.

Although several studies have reported beneficial actions of activation or reactivation of PPARα signalling on the development and progression of LVH, this remains a controversial issue with other studies reporting limited benefit of PPARα agonists on cardiac hypertrophy or an aggravated hypertrophic response following PPARα activation. For example, PPARα activation with fenofibrate in hypertensive rats failed to prevent cardiac hypertrophy, although associated cardiac fibrosis and LV chamber dilatation were reduce [48,49]. Furthermore, in rats subjected to pressure overload induced by abdominal aortic constriction for 7 days, PPARα activation resulted in a significantly worsened cardiac hypertrophy and systolic dysfunction, compared to untreated control animals [50]. The complexity of PPARα activation is further highlighted by the observation that fenofibrate exerts beneficial cardiac effects in WT mice subjected to abdominal aortic constriction, but exacerbates adverse cardiac remodelling, contractile dysfunction and mortality in PPARα^−/−^ mice, suggesting that fenofibrate may exert PPARα-independent effects in this setting [51]. Interestingly, another study using spontaneously-hypertensive rats reported that fenofibrate protected against cardiac hypertrophy during the initial stages of hypertension (2 months), but with prolonged hypertension (6 months) the cardiac hypertrophic phenotype was aggravated by drug treatment [52], highlighting temporal considerations with regard to PPARα activation. Nonetheless, in the present study we confirmed PPARα as a key regulator of cardiac hypertrophy and dysfunction in response to pressure overload, which influences both myocardial Nox2 expression and redox status.

Taken together, our in vitro and in vivo data are clearly supportive of emerging evidence highlighting cross-talk between PPARα and Nox2 as an important regulatory axis in CHF—the precise nature of which has not been previously defined in this setting. However, it is important to note that similar interaction has been observed with acute myocardial ischaemia/reperfusion injury, which was reported to be worsened by suppression of NADPH oxidase-derived ROS and consequent PPARα upregulation and HIF-1α downregulation [30] Nonetheless, it is well established that NADPH oxidase signalling in the context of cardiac remodelling is particularly dependent on stress, with previous work highlighting significant responses to, for example e.g., myocardial infarction, angiotensin II infusion, and doxorubicin cardiotoxicity [2,5,43] As such, our data highlight novel directional interaction between Nox2 and PPARα as a key driver of cardiomyocyte remodelling in response to pressure overload, which is quite different to acute myocardial ischaemia/reperfusion injury, whilst defining specific pathways likely to mediate maladaptive signalling in this setting. In this regard, detailed transcriptomic analysis of hearts from WT, PPARα^−/−^ and Nox2^−/−^ mice subjected to TAC revealed that oxidative stress pathways remain dysregulated in the absence of PPARα with limited overlap in biological signalling observed in the absence of Nox2, indicating predominant directional regulation by Nox2. It is not surprising that such interaction appears to be largely mediated via Nox2-dependent modulation of immune and inflammatory pathways, which is reported to suppress regulatory T cells thereby drive adverse cardiac remodelling [51,52,53,54,55,56], resulting in downregulation of key metabolic pathways linked with PPARα signalling. Nonetheless, it is interesting that detailed IPA, focussing on inflammatory pathways predominantly altered in WT and PPARα^−/−^ hearts, identified several conserved and differentially-activated cytokines as candidate upstream mediators of the TAC response—several of which have been previously linked with PPARα signalling (e.g., IL-1β, IL-6, CCL2, CCL5, and CXCL2) in cardiac and non-cardiac settings [57,58,59]. Furthermore, subsequent network analysis strongly predicted Nox2 as an upstream master regulator of inflammatory signalling in response to TAC, specifically highlighting six genes as likely targets—three of which (IL-1β, IL-6, CCL5) were also implicated in the previous IPA and associated with PPARα signalling. It is particularly notable that this analysis identified several predicted intermediate regulators of Nox2-dependent cytokine activation in TAC, including p38MAPK, JNK, ERK1/2, AP-1 and NF-κB, which are known to mediate cardiac ROS generation and Nox2 signalling [38], providing further confidence in our implication of Nox2 as a critical driver of PPARα downregulation and central pathway promoting adverse cardiac remodelling and dysfunction in response to pressure overload.

In summary, the data presented in this study confirm previous reports of key independent roles for Nox2 and PPARα in regulating the development and progression of pressure overload-induced LVH, whilst demonstrating for the first time direct interaction between these two proteins which is critically dependent upon Nox2 regulation of inflammatory signalling networks. In addition, this study has implicated several cytokines as likely mediators of novel Nox2-PPARα directional interaction underlying maladaptive cardiac signalling in response to TAC. It, therefore, seems plausible that such candidates may represent potential pharmacological targets to support more effective clinical treatment of CHF, although further investigation is clearly required to confirm roles of specific cytokines and to define detailed inflammatory pathways underlying Nox2-dependent PPARα signalling in this setting.

## 4. Materials and Methods

### 4.1. H9c2 Cardiomyoblast Culture

H9c2 cardiomyoblasts (ATCC, Manassas, VA, USA) were cultured in low-glucose (5.5 mmol/L) Dulbecco’s Modified Eagle’s Medium (DMEM; Sigma-Aldrich, St. Louis, MO, USA) supplemented with 10% foetal calf serum (Sigma-Aldrich), 100 units/mL penicillin and 100 μg/mL streptomycin (Fisher Scientific, Loughborough, UK) at 37 °C and 5% CO_2_ [60]. Prior to stimulation, cells were seeded in 12-well plates at a density of 12,000 cells/well and incubated in 0.5% serum-containing DMEM for 24 h to induce quiescence. Cell hypertrophy was then stimulated by exposure to phenylephrine (PE; 10 μmol/L) for 24 h in the presence or absence of the pan-NADPH oxidase inhibitor, VAS2870 (10 μmol/L, Vasopharm, Würzburg, Germany), to dissect the specific contribution of Nox-derived ROS [61], or fenofibrate (10 μmol/L, Sigma-Aldrich), to identify the involvement of PPARα in H9c2 hypertrophy [62]. VAS2870 treatment of H9c2 cardiomyocytes completely abolished PE-induced increases in NADPH-dependent superoxide production (Figure 1F), whilst fenofibrate treatment of H9c2 cardiomyocytes increased protein expression of PPARα protein expression (Figure S2), demonstrating efficacy in our employed culture model. For analysis of morphometric hypertrophy, cells were washed twice with PBS and adherent cells were fixed with 10% neutral buffered formalin (Sigma-Aldrich) prior to staining with 0.1% crystal violet (Sigma-Aldrich) for 10 min [63]. Cells were then rinsed by gentle submersion in water and briefly air-dried before being imaged on an inverted phase-contrast microscope (Nikon Eclipse, Tokyo, Japan). Cell area was quantified in 3 wells/treatment condition analysing 6 fields/well, excluding any cells undergoing division or exhibiting abnormal nuclear morphology, membrane blebbing or any other degenerative morphological features. Analysis was performed in a blinded manner (NIS Elements, Nikon, Tokyo, Japan) using a polygon tool to assess a minimum of 40 cells per treatment group and data for the control groups normalised to 1 in order to facilitate comparison of relative cell area across treatments.

### 4.2. Experimental Animals

Experiments were performed using previously characterised gene-modified mice with global deficiency of Nox2 (Nox2^−/−^) or PPARα (PPARα^−/−^) with comparison to WT C57BL/6J littermate controls (8–12 weeks; The Jackson Laboratory, Bar Harbor, Maine, USA) [15,44]. All studies were performed in accordance with the Guidance on the Operations of the Animals (Scientific Procedures) Act 1986 (UK) and approved by the Queen’s University Belfast Animal Welfare and Ethical Review Body under the authority of Project Licence 2714 issued on 10 January 2017 by the Department of Health (Northern Ireland). Animals were group-housed under standard conditions (12 h light–dark cycle, 21 °C, ad libitum food/water).

### 4.3. Transverse Aortic Constriction

Mice were randomised prior to induction of pressure overload cardiac hypertrophy by minimally-invasive TAC [64] under 2% isofluorane/oxygen anaesthesia (with pre/post-operative buprenorphine analgesia, 0.05 mg/kg i.m., as required) or sham surgery, which involved an identical procedure with the exception of aortic constriction. Briefly, a small horizontal skin incision (5–10 mm) was made at the level of the suprasternal notch to locate the trachea, before a 5 mm longitudinal cut was made along the sternum. The thymus gland was then retracted and the aortic arch isolated before a 5/0 silk suture was passed under the vessel between the origin of the right innominate and left common carotid artery. An angled 27 gauge needle was then placed beside the vessel and the suture firmly tied around both the vessel and needle before the latter was quickly removed. After TAC or sham surgery, the skin was sutured with 5/0 vicryl and mice were kept in a heated recovery chamber for up to 24 h before being transferred back to standard housing.

### 4.4. Echocardiography

After 7 days, mice were anaesthetised with 1.5% isofluorane/oxygen, placed on a warming pad with ECG measurement (for assessment of heart rate), and imaged in the supine position using a Vevo770 ultrasound system with high-frequency 45 MHz RMV707B scanhead (VisualSonics, Amsterdam, The Netherlands). M-mode parasternal short-axis scans at the level of the papillary muscle were used to quantify LVEDD and LVESD, from which % fractional shortening was calculated using the equation (LVEDD−LVESD)/LVEDD × 100. Pulse-wave Doppler was used to assess mitral valve flow (E/A ratio), LV isovolumetric relaxation time and myocardial performance index, as reliable measures of diastolic function. Images were quantified by the same observer in a blinded manner in order to minimise variability and bias.

### 4.5. Cardiac Morphometry and Cardiomyocyte Hypertrophy

After functional analysis, animals were sacrificed by cervical dislocation and the heart was rapidly excised and measurements of whole heart and LV weight taken and indexed to body weight. For assessment of cardiomyocyte hypertrophy, hearts were arrested in diastole by intracardiac infusion of 1 M KCl in order to standardise structural and histological measurements which were performed using paraffin-embedded LV sections (5 μm). Cardiomyocyte cross-sectional area was determined by H&E staining, analysing cells with centrally located nuclei; 5–6 random sections per heart were assessed, using 5 separate fields in each, and data quantified by blinded digital image analysis (NIS-Elements, Nikon).

### 4.6. Quantitative RT-PCR

Total RNA was extracted from cells or LV homogenate using TRI reagent (Sigma-Aldrich), and cDNA synthesised by reverse transcription (Applied Biosystems, Foster City, CA, USA). mRNA expression was then quantified by real-time RT-PCR using fluorescent SYBR green (Prism 7300, Applied Biosystems) and commercially-designed primer sets (Qiagen, Hilden, Germany) with GAPDH or 18S used for normalisation (whose expression was shown to remain unaltered between experimental groups in both cells and tissues). Quantification was performed using the comparative Ct method [2].

### 4.7. Lucigenin-Enhanced Chemiluminescence

NADPH-dependent superoxide production was assessed using lucigenin-enhanced chemiluminescence [1,5]. Briefly, cells or freshly-dissected LV (approximately 30 mg) were lysed in Buffer B (50 mmol/L monobasic potassium phosphate, 1 mmol/L EGTA, 150 mmol/L sucrose) with protease inhibitor cocktail (Roche, Basel, Switzerland) prior to sonication for 3 × 10 s on ice. Following centrifugation, the supernatant was removed and pellets re-suspended in Buffer B with protease inhibitor cocktail, before quantification using the Bradford assay. Protein (50 μg) was then loaded in to white 96-well plates before the superoxide reaction was initiated by adding 25 μL NADPH (1 mg/mL; Sigma-Aldrich) to each sample and incubating for 10 min at 37 °C. An initial fluorescence reading was taken before 25 μL lucigenin (10 μmol/L; Sigma-Aldrich) was added to each well and serial measurements taken every 60 s for 30 min (Berthold Tristar). Superoxide production was quantified as area under the curve, calculated by subtracting the mean background reading from each mean sample value. All experiments were performed in triplicate with mean superoxide levels presented in relative light units.

### 4.8. Determination of Protein Carbonylation

An Oxyblot protein oxidation detection kit (Merck Millipore, Burlington, MA, USA) was used to determine the degree of protein carbonylation in LV samples, following the manufacturer’s protocol and as previously described. Protein lysate (50 µg prepared in lysis buffer containing 50 mmol/L Na_4_P_2_O_7_, 50 mmol/L NaF, 50 mmol/L NaCl, 5mmol/L Na_2_EDTA, 10mmol/L Hepes, 0.5% (*v*/*v*) triton X-100, pH 7.4; 1 mmol/L Na_3_VO_4_, 1 mmol/L PMSF, 2 tablets of complete EDTA-free protease inhibitor cocktail per 50 mL), were treated with 50 mmol/L DTT. Samples were then mixed with 12% SDS (1:1); one part (25 µg) was treated with DNPH derivatisation solution (1:1) and the other part (15 µg) with negative control solution (1:1) before incubation for 15 min at room temperature. The reactions were then stopped by addition of neutralisation solution and samples directly loaded onto a polyacrylamide gel for electrophoresis and subsequent immunoblotting.

### 4.9. Gene Array Analysis

Microarray analysis of RNA samples from mouse LV tissue (*N* = 3–4 biological replicates) was conducted by the Queen’s University Belfast Genomics Core Technology Unit using an Illumina MouseRef-8 v2.0 Expression BeadChip Kit run on an Illumina-approved bead array system. Briefly, RNA samples were amplified and labelled using an Illumina total prep RNA amplification kit where samples were reverse transcribed to synthesise first strand cDNA followed by second strand cDNA synthesis and purification. In vitro transcription resulted in generation of biotinylated cRNA which was dispensed onto a bead chip consisting of microwells containing oligonucleotides immobilised to beads randomly distributed across the surface of the array. This was then placed onto a rocking platform in an Illumina hybridisation oven set at 58 °C to hybridise samples onto the beadchip and incubated overnight. Following sample preparation and hybridisation, beadchips were washed before blocking for 10 min with E1 blocking buffer (Illumina) and placing into a wash tray containing streptavidin-Cy3 and rocked at medium speed for 10min to facilitate binding to biotinylated cRNA before a final wash. The beadchip was then dried and loaded into the Illumina approved bead array system in which bead location was decoded using the decoding algorithm utilising specificity and reversibility of DNA hybridisation [65]. Raw gene expression data were normalised using Lumi Bioconductor R package and imported into Partek Genomics Suite v6.6 for visualisation using principal component analysis and differential gene expression analysis using analysis of variance (ANOVA). Data were filtered prior to analysis using median variance. Gene lists were generated using unadjusted *p*-values (*p* < 0.05) and a fold change of ±1.5. Gene set enrichment analysis (GSEA; Broad Institute, UC San Diego, San Diego, CA, USA) data were downloaded at http://software.broadinstitute.org/gsea/index.jsp and gene sets BIOCARTA_PPARA_PATHWAY, GO_RESPONSE_TO_OXIDATIVE_STRESS and KEGG_HYPERTROPHIC_CARDIOMYOPATHY were downloaded from the molecular signatures database (MsigDB) at http://software.broadinstitute.org/gsea/msigdb/index.jsp. Pathway enrichment analysis and upstream regulator analysis were performed using IPA software. R (v3.6.3) was used to visualise IPA outputs.

### 4.10. Statisical Analysis

All in vitro, tissue or functional data were analysed using GraphPad Prism software including identification of statistically significant outlier values using Grubb’s test. Data were compared using one-way analysis of variance (ANOVA) with Bonferroni post-hoc testing for multiple groups or unpaired Student’s t-test for two groups. In all cases, *p* < 0.05 was considered to indicate statistical significance. Gene array data were subjected to appropriate statistical analysis as detailed in the previous methods section.

## Supplementary Materials

Supplementary materials can be found at https://www.mdpi.com/1422-0067/21/12/4406/s1.

## Abbreviations

CHF	Chronic heart failure
DEG	Differentially expressed gene
GSEA	Gene set enrichment analysis
IPA	Ingenuity pathway analysis
LVEDD	Left ventricular end-diastolic diameter
LVESD	Left ventricular end-systolic diameter
LVH	Left ventricular hypertrophy
PE	Phenylephrine
PPARα	Peroxisome proliferator-activated receptor-α
ROS	Reactive oxygen species
TAC	Transverse aortic constriction
WT	Wild-type
