# Supplementary material for: Downregulation of PPARα during Experimental Left Ventricular Hypertrophy is Critically Dependent on Nox2 NADPH Oxidase Signalling
Source: Int J Mol Sci. 2020 Jun 20;21(12):4406. doi: 10.3390/ijms21124406 (PMC7352162; doi:10.3390/ijms21124406)
Supplement: Supplementary file 1 [file ijms-21-04406-s001.pdf]

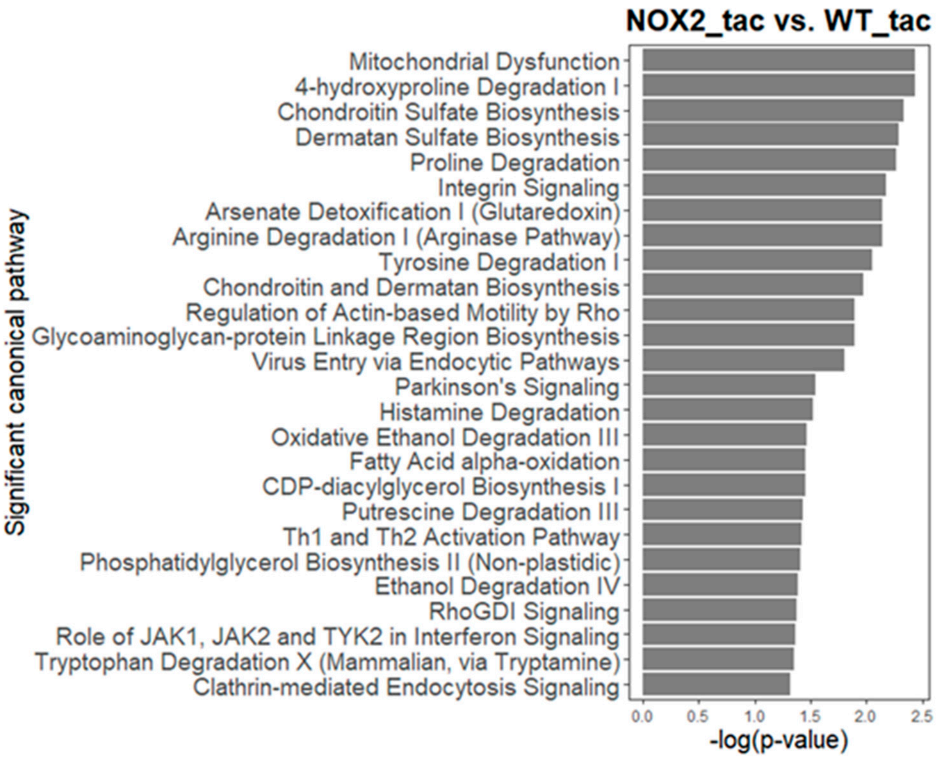

Supplementary Figure 1. Canonical pathway analysis of differentially expressed genes using IPA. Significantly enriched pathways in Nox2$^{-/-}$ TAC vs WT TAC mice (grey indicates that no activation z-score could be predicted by IPA). N=3-4.

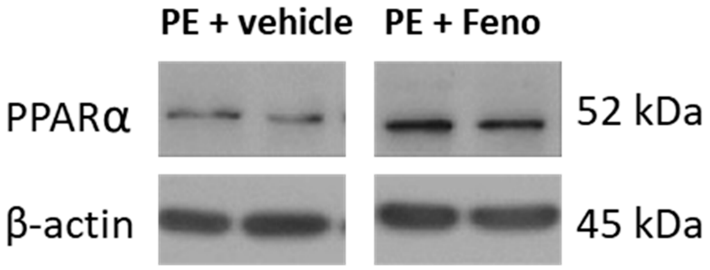

Supplementary Figure 2. Fenofibrate treatment activates PPAR in H9c2 cardiomyocytes. Cells were incubated with vehicle or the PPARα agonist fenofibrate (10µmol/L) prior to PE treatment for 24h and PPARα protein expression analysis by western blot.
